# Management of instability after primary total knee arthroplasty: an evidence-based review

**DOI:** 10.1186/s13018-021-02878-5

**Published:** 2021-12-20

**Authors:** Talal Al-Jabri, Angela Brivio, Nicola Maffulli, David Barrett

**Affiliations:** 1 Trauma and Orthopaedic Surgery, Department of Surgery and Cancer, Imperial College London, London, SW7 2AZ England; 2 King Edward VII’s Hospital, 5-10 Beaumont Street, Marylebone, London, W1G 6AA England; 3 Department of Trauma and Orthopaedic Surgery, Istituto Clinico Città Studi, Milano, Via Niccolò Jommelli, 17, 20131 Milano, MI Italy; 4 Department of Medicine, Surgery and Dentistry, University of Salerno, Via S. Allende, 84081 Baronissi, SA Italy; 5 Barts and the London School of Medicine and Dentistry, Centre for Sports and Exercise Medicine, Mile End Hospital, Queen Mary University of London, 275 Bancroft Road, London, E1 4DG England; 6 School of Pharmacy and Bioengineering, Keele University School of Medicine, Stoke on Trent, ST5 5BG UK; 7 Spire Hospital, Southampton, SO16 6UY UK; 8 School of Engineering Sciences, University of Southampton, Highfield, Southampton, SO17 1BJ UK

**Keywords:** Recurvatum, Flexion, Extension, Mid-flexion, Knee Replacement, Instability, Revision

## Abstract

**Background:**

Instability is one of the most common reasons for revision after a total knee replacement. It accounts for 17.4% of all single-stage revision procedures performed in the UK National Joint Registry. Through a careful patient evaluation, physical assessment and review of investigations one can identify the likely type of instability.

**Aims:**

To critically examine the different types of instability, their presentation and evidence-based management options.

**Method:**

A comprehensive literature search was conducted to identify articles relevant to the aetiology and management of instability in total knee replacements.

**Results:**

Instability should be categorised as isolated or global and then, as flexion, mid-flexion, extension or recurvatum types. By identifying the aetiology of instability one can correctly restore balance and stability.

**Conclusion:**

With careful judgement and meticulous surgical planning, instability can be addressed and revision surgery can provide patients with successful outcomes.

## Background

The UK National Joint Registry (NJR) is the largest registry of its kind in the world and has recently been described by the British Under Secretary of State for Health and Social care as ‘a global exemplar’ for foreign registries [1]. A total 1,145,050 total knee replacements (TKRs) are recorded in it, with an overall revision rate at 15 years of 4.75% for cemented TKRs [1]. In the USA, by 2030 demand for primary TKRs has been projected to grow by 673% to 3.48 million procedures per year given the ageing population and increased incidence of osteoarthritis [2]. In accordance with this, the number of primary TKRs and consequent revision procedures performed in the UK annually has risen over the last decade with 6708 TKR revision procedures performed in 2019 compared to 4393 procedures in 2008 [1]. 65,377 single-stage revisions in total have been recorded in the UK NJR, with 11,397 (17.4%) of these being performed to address instability [1].

Over the last 2 decades, instability has been identified as the underlying cause for 10 to 26% of revision procedures [2–6]. Additionally, multiple studies have revealed that only 82% to 89% of patients are satisfied after their primary TKR, and while there are almost certainly multiple factors contributing to this, instability, one of the most common reasons for revision, still is an often underappreciated culprit for dissatisfaction [7–10].

This article provides an overview of the different types of instability, their causes and evidence-based management options.

## Methodology

We conducted a review of the literature with a defined search strategy to identify articles relevant to instability in TKR. This included a search on MEDLINE and Google Scholar from January 2000 to September 2021. Search terms included (‘total knee arthroplasty’ [All fields] OR ‘total knee replacement’ [MeSH terms] with all entry terms and ‘instability’ [All fields]) OR (‘unstable’ [MeSH terms]). Journals in all languages were included with no limits in the search strategy. Abstracts were screened for relevance. Exclusion criteria included letters, editorials and studies recognised as being of poor design or of a low level of evidence. The references of the selected full text articles were reviewed for the inclusion of additional articles. All selected articles were critically appraised by the authors.

### Classification and patient evaluation

There are different types of instability, which is defined as abnormal and excessive displacement of the prosthetic joint which leads to its failure [4]. A broad classification of instability comprises 3 main types: flexion instability, genu recurvatum and extension instability [4]. Kelly Vince and colleagues described 4 types of instability, namely varus–valgus, recurvatum, anterior–posterior (AP)/flexion instability and global/gross instability [11]. Most of the modern literature supports classifying instability pragmatically as [12, 13]:

1. Extension instability
2. Genu recurvatum
3. Flexion instability
4. Mid-flexion instability
5. Global multiplanar instability

Additionally, the temporal behaviour is often noted, with acute instability presenting in the first few months postoperatively and chronic instability occurring beyond this early period. Attention to this during history-taking provides clues as to the aetiology of instability. Acute instability present immediately postoperatively may be indicative of component malalignment, failure to balance the flexion–extension gaps or to achieve satisfactory alignment. A period of reasonable satisfaction postoperatively which is suddenly followed by instability may occur secondary to attenuation or rupture of the posterior cruciate ligament (PCL) or collateral ligaments. This can be of particular concern when pie-crusting and other soft tissue releases have been performed at the index procedure. Therefore, one must have a detailed report of the primary procedure. There are multiple reasons for late instability, but polyethylene wear and/or late ligamentous dysfunction is typical aetiologies. During revision procedures it is not uncommon to identify medial wear: this indicates malalignment of components which, over time, can lead to varus deformities and instability [12].

During patient evaluation, clinicians must think about the commonest causes of instability and whether they are present. A useful framework to categorise the aetiologies includes:

- **Patient-specific factors** (e.g. traumatic falls postoperatively, a history of connective tissue disorders or neuromuscular disorders such as poliomyelitis).
- **Implant-related factors** (e.g. implant type and design, wear and bone loss/osteolysis causing loosening/settling of implants and a progressive gap imbalance and instability).
- **Technique-specific factors** (malalignment of the implant, malrotation, ligament failure or attenuation, inadequate/inaccurate bone resections and failure to balance the knee coronally through either under-release or inadvertent over-release of the soft tissue envelope).

History taking should focus on:

- Details of the initial TKR including the date, prosthesis and indication for the index procedure, previous surgical history to the knee and limb, a record of any pre-operative malalignment or contractures/deformities, operative details of the strategies employed to treat any deformities, information regarding the bone resections performed and gap balancing assessments intraoperatively. Any intra-operative or postoperative complications should be sought.
- Fare one must determine the presence of pain and look for a history of wound complications and features of periprosthetic joint infection (PJI) such as night pain, pyrexia or an effusion. In all cases, it is prudent to follow the most recent guidance for the diagnosis of PJI provided by the Musculoskeletal Infection Society (MSIS) [14]. This will help exclude infection and provide clinicians with a greater degree of diagnostic certainty when considering instability as the cause for the patients dissatisfaction. The nature, onset and timing of pain may vary between the types of instability. For example, pain at rest or with extension may indicate a tight and overstuffed extension gap or patellofemoral compartment in the absence of infection. A sudden pop accompanying a feeling of localised pain may indicate late ligamentous rupture. Pain upon weight bearing may indicate component loosening or malalignment.
- The nature of the instability and whether it is progressively worsening should be ascertained. Patients with flexion instability may typically complain of the knee ‘giving way’ upon descending stairs or ‘not being able to trust the limb on uneven surfaces’. Rising from chairs commonly manifests symptoms of instability [15].
- Patients may also complain of recurrent effusions with instability and abnormalities in their range of motion. Flexion instability patients typically have a good range of motion in the early postoperative period as their flexion gaps are loose, and they may present dramatically with a cam-post dislocation in posterior stabilised TKRs.
- The co-morbidities of the patient can also expose likely types of instability such as genu recurvatum in poliomyelitis or global instability in Ehlers-Danlos syndromes (EDS).

A thorough clinical examination should be performed. This should not just be limited to the knee and instead includes an assessment of any deformities at the ankle or hip which may affect overall limb alignment. The fluidity of the gait pattern should be examined with distinct appreciation of any varus or valgus thrust (present in asymmetric extension instability). Malrotation of the femoral component may manifest in an abnormal foot progression angle during gait and patellar mal-tracking. This too creates an asymmetric, trapezoidal-shaped flexion gap and flexion instability. The knee should be systematically palpated for evidence of localised tenderness (e.g. pes anserinus tendonitis and/or iliotibial band (ITB) tendonitis which can occur in flexion instability) and an effusion. It is not uncommon for patients to present with a serosanguinous effusion. This has been reported to occur in 70% to 85% of patients with flexion instability [15, 16].

The range of motion should be noted as this varies significantly between the types of instability encountered. For example, a knee which fails to fully extend may occur secondary to a tight extension gap. This can ultimately lead to a flexion contracture of the TKR. Conversely, laxity in extension produces recurvatum. Tightness in flexion will limit the amount of deep flexion achieved if any [16, 17]. Concomitantly, an assessment of patellofemoral tracking should be performed. We routinely perform varus–valgus stress testing at full extension, 30°, 45°–60° and 90° of flexion to appreciate the condition of the surrounding knee stabilisers. Instability is a clinical diagnosis and should not be overlooked or neglected during patient assessment. Specific clinical findings for flexion instability are reported below:

- A positive posterior sag sign on inspection can be observed as the flexion gap opens during knee flexion in cruciate retaining (CR) prostheses [18]. A posterior stabilised (PS) implant may not present with a ‘sag sign’. However, flexion instability in these implants is often associated with collateral ligament laxity indicating the importance of varus–valgus stress testing [4]. In the absence of a posterior sag, an increased anterior draw was indicative of flexion instability in PS TKRs [18].
- Flexion instability typically presents with a positive anterior draw at 90° of flexion, with translation classified as mild (< 5 mm (mm)), moderate (< 10 mm) and marked (> 10 mm). However, an agreement on the amount of translation which is diagnostic of instability has not been reached [16, 17]. In our experience, marked translation is highly indicative of flexion instability. AP translation should also be assessed at full extension and mid-flexion, especially when considering mid-flexion instability as a diagnosis.
- Vince described how flexion instability can be visualised during inspection. They suggested seating the patient on the edge of the examination table to allow the knee to flex to approximately 90° and for the quadriceps to be relaxed. The weight of the limb will allow for the flexion space to open up, which can be acknowledged by the astute examiner. The patient is subsequently asked to extend the knee, which will cause the tibia to be ‘pulled up’ as the quadriceps contracts to re-establish contact between the tibial liner and the femoral component after which extension occurs [19].

To complete the patient evaluation a series of diagnostic studies should be undertaken including weight-bearing AP, lateral and skyline knee radiographs, full-length coronal and sagittal radiographs are obligatory to assess mechanical or anatomical alignment of the TKR. Computed tomography (CT) will establish the presence of malrotation but can also provide further information regarding osteolysis, loosening and residual bone stock. Lateral radiographs in full extension, 90° of flexion and then maximum flexion can be performed to illustrate the flexion and extension gaps. It is useful to compare the images obtained with pre-operative and early postoperative radiographs to help identify any changes occurring to develop an understanding of the likely prognosis for the patient [20]. Where deformities are obvious a varus–valgus stress radiograph may confirm whether these are reducible and illustrate the integrity of the soft tissue envelope. It is however worth stressing that there is limited evidence to guide the interpretation of stress views [21].

### Extension instability

**Fig. 1:**
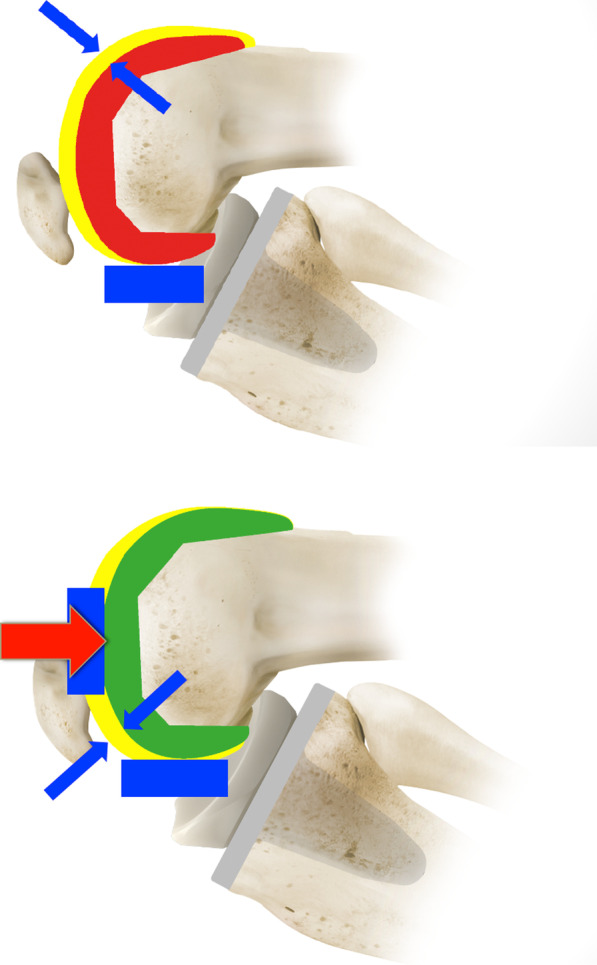
This illustration shows the affect of over-resection of the distal femur on third space under-stuffing and mid-flexion instability

Extension instability is sub-classified as symmetric instability or the commoner asymmetric instability. Yercan et al. also coined the expression ‘instability due to bone resection’ when describing symmetric extension instability in recognition of the fact that an excessive distal femoral cut can lead to this [22, 23]. In the presence of a knee which is balanced in flexion but loose in extension, one can appreciate a degree of hyperextension when assessing the range of motion accompanying the elevated joint line. Pre- and postoperative radiographs must be scrutinised to determine the height of the joint line against anatomical landmarks [22]. Excessive bone removal from the tibia will increase the flexion and extension gaps equally, and so adequate filling can be achieved by inserting a thicker tibial insert. In excessive distal femoral resection alone a thicker tibial insert would raise the joint line and cause flexion gap tightness limiting deep flexion. Joint line elevation would also result in a pseudo-patella baja, impaired patellofemoral biomechanics and a loss of isometry in the surrounding knee ligaments which could contribute to mid-flexion instability [11, 19, 22]. In this situation, symmetric extension instability is best treated using distal femoral augments added to the prosthesis to restore the correct joint line height, balance the extension gap and correct patellofemoral under-stuffing (Fig. 1) [4].

Incomplete correction of a coronal plane deformity in extension or unrecognised iatrogenic injury to the collaterals leads to asymmetric extension instability [19, 22, 23]. It is not uncommon for surgeons to under-correct a deformity out of fear of causing ligamentous instability, and ultimately having to convert to a more constrained implant [19]. However, in pre-operative varus knees an under-correction of the tight medial compartment over time will predispose to varus component malalignment and/or facilitate a residual postoperative varus deformity. The tight medial structures will lead to more medial polyethylene wear and a progressive varus upon weightbearing. The lateral structures will stretch over time, and the extension gap will become progressively more trapezoidal and unbalanced. This situation is familiar to experienced revision knee surgeons: in 1987 Laskin et al. showed that under-correction of a fixed varus knee deformity in four of their 68 patients led to asymmetric extension instability [24]. The superficial medial collateral ligament (MCL) tends to be the major deforming force in these knees, and several authors described various techniques to achieve coronal balance [4, 25]. Insall’s legacy in knee arthroplasty is remarkable, and his 1985 paper still stands, with many surgeons adopting his technique for medial release [26]. This involved externally rotating the foot to avoid release of the pes anserine tendons and a subperiosteal release of the superficial MCL at its tibial insertion [26, 27]. Whiteside recommended that the part of the ligament to be released should vary according to whether the asymmetry is identified in flexion or extension. He suggested that medial tightness in flexion is best treated by releasing the anterior fibres of the superficial MCL, whereas release of the posterior fibres would correct medial tightness in extension [22, 27]. Both portions should be released if tight in flexion and extension [27]. The technique developed at the Hospital for Special Surgery in New York addressed varus and fixed flexion deformities: they showed reduced the risk of over-release, postoperative haematomas or the need for constrained implants [28]. The technique involves making a posteromedial capsulotomy at the level of the tibial bone cut and ‘pie-crusting’ the MCL in extension followed by serial manipulations with valgus stress [28]. Our preferred technique is to undertake a sequential release of the medial structures starting with a subperiosteal release of the deep MCL from the tibial tuberosity to the posteromedial corner of the tibia, removing medial osteophytes, considering a release of the semimembranosus when a combined flexion contracture is present and then addressing the superficial MCL if instability persists.

Valgus knees pose a particular challenge to arthroplasty surgeons primarily because these are less commonly encountered than varus knees, and the course of the common peroneal nerve adds to the risk and complexity of performing releases. Multiple approaches to dealing with valgus knees have been proposed, with the first of these being the technique described by Insall et al. [26]. They originally described a series of releases commencing with the lateral collateral ligament (LCL) then, the popliteus tendon, lateral head of the gastrocnemius and ending in the ITB if an external rotation deformity was present [4, 26]. Laskin et al. recommended a similar series of releases, but differed from Insall et al.’s sequence in that he would release the ITB first followed by a step-wise release of the remaining structures. Favorito et al. were also in favour of releasing the LCL first as he felt that this was the tightest structure contributing to the deformity [29]. These two early techniques unfortunately led to over release of ligaments and reports of instability and even dislocations with posterior stabilised implants [4, 30]. When addressing this deformity it is important to understand that there are osseous and soft tissue elements producing it. Often there is a hypoplastic lateral femoral condyle that needs to be accounted for when making the bone cuts. Whiteside suggested that lateral tightness in flexion primarily resulted from the LCL and popliteus tendon, while in extension the ITB and posterolateral capsule were primarily responsible. Based on this, targeted releases in a series 239 knees resulted in no cases of clinical instability [27].

Insall’s original technique was later modified to recommend ‘pie-crusting’, which is now common practice [31, 32]. This involves inserting and opening a lamina spreader in the extension gap of the knee when in full extension. A transverse incision is made through the posterolateral capsule posterior to the LCL and anterolateral to the popliteal tendon. The lateral extension gap is then increased by making a series of horizontal stab incisions into any structure that feels tight. By doing this and regularly assessing the extension gap over-release is unlikely to occur. Additionally, it is critical to limit the depth of the insertion of the blade to less than 5 mm as this reduces the risk of inadvertent injury [31, 32]. Clarke et al. reported no cases of instability with this technique in 24 valgus knees with a mean pre-operative valgus of 15° [31]. When deformities exceed 20° of valgus, concern regarding stretching of the common peroneal nerve led Easley et al. to consider under-correction, though this can be controversial. Easley et al. suggested using a varus–valgus constrained implant to provide stability for the MCL which would not be taut in under-correction. They reported no cases of common peroneal nerve palsy or cases of instability with an average follow-up of 7.8 years in 28 knees [33]. This technique is considered controversial given the risk of aseptic loosening with a more constrained prosthesis and is generally not recommended in more active patients [4, 33].

**Fig. 2:**
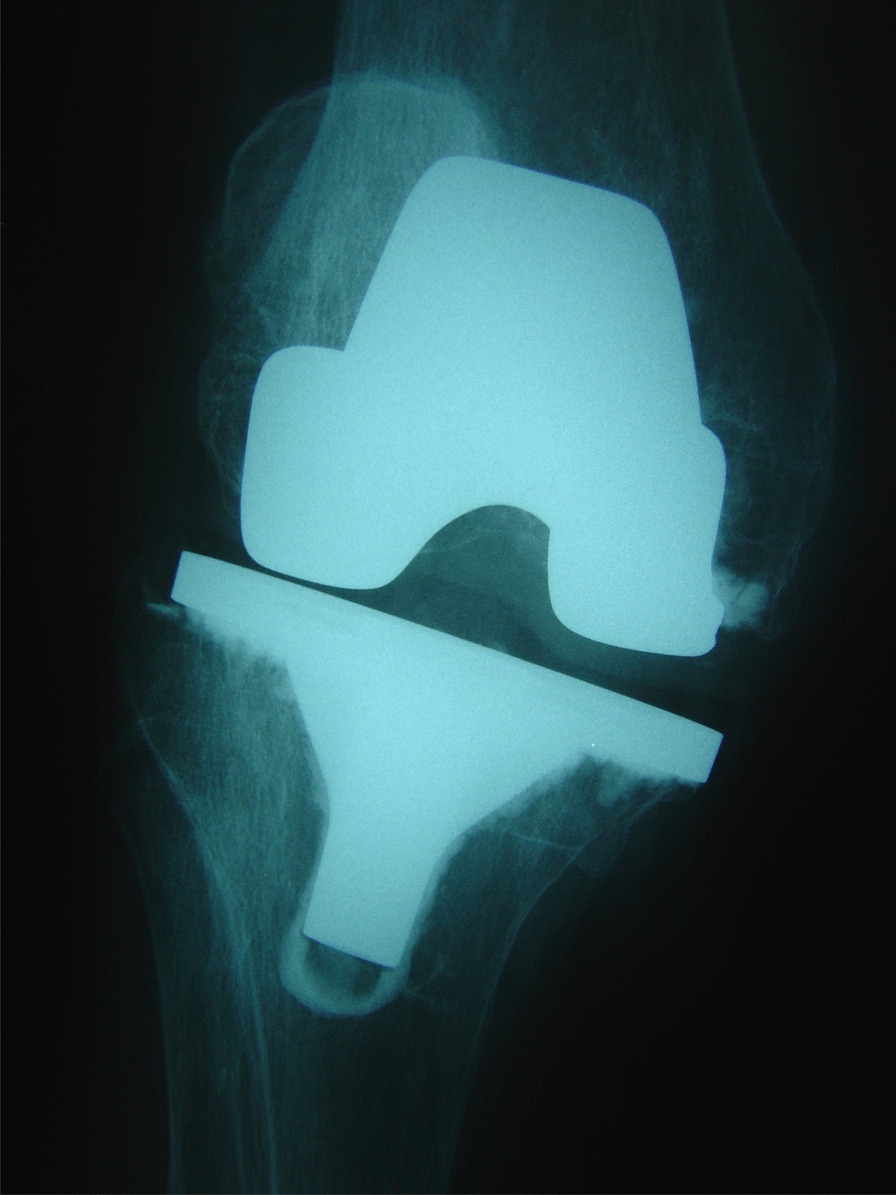
This AP radiograph demonstrates significant laxity to valgus stress due to malalignment of the tibial component which ultimately required revision

When dealing with extension instability, one must remain vigilant to the possibility of iatrogenic collateral ligament injury when performing the tibial resection or during varus–valgus stress testing (Fig. 2). If this occurs, surgical options include re-approximation of the torn ends with a Krackow-type suture, hamstring autograft reconstruction and to augment a repair and one must consider whether a varus–valgus constrained implant is required [34]. In a series of 600 knees there were 16 MCL disruptions treated with direct primary repair or suture-anchor reattachment to bone, followed by the use of a hinged knee brace for 6 weeks postoperatively. No patient required revision to a varus–valgus constrained implant, with all patients reporting good or excellent outcomes [35].

### Genu recurvatum

This is extremely rare, and pre-operatively < 1% of all patients presenting for TKR have this deformity [4]. It tends to occur in patients who suffer from a neuromuscular disorder such as poliomyelitis, but can occur in patients with a valgus deformity and a tight ITB or in cases of cruciate and collateral ligament laxity such as in rheumatoid arthritis [4]. During pre-operative evaluation, it is vital to assess the strength of the quadriceps muscle, as patients with significant weakness will stabilise their knee while standing by forcing it into hyperextension. Although these are challenging conditions, surgical options have been proposed and include the use of distal femoral augments or under-resection of the distal femur to tighten the extension gap when using an unconstrained implant, transfer of the femoral origins of the collateral ligaments proximally and posteriorly to allow for tightening during extension, or the use of a rotating hinged implant with an extension stop [4, 36, 37].

### Flexion instability

Flexion instability was first described by Pagnano et al. in 1998. However, when speaking at the international congress for joint reconstruction Dr Arlen D. Hanssen described how he and his colleagues became familiar with flexion instability in 1993. He described a large number of ‘unhappy knees’ primarily from flexion instability; however, the recurrent effusions that are typical in these patients would engender diagnostic problems for less familiar surgeons [38, 39]. Flexion instability has unfortunately remained a diagnostic challenge, though it does present with a typical constellation of signs (recurrent serosanguinous effusions, pes anserine/ITB tendinopathy, instability rising from chairs or ascending/descending stairs and a positive anterior draw) [16, 39]. The causative factor is a flexion gap which exceeds that of the extension gap which can occur due to:

- An undersized femoral component
- Rupture of the PCL in CR TKRs
- An excessive posterior sloped tibial cut
- Excessive resection of the posterior femoral condyles

**Fig. 3:**
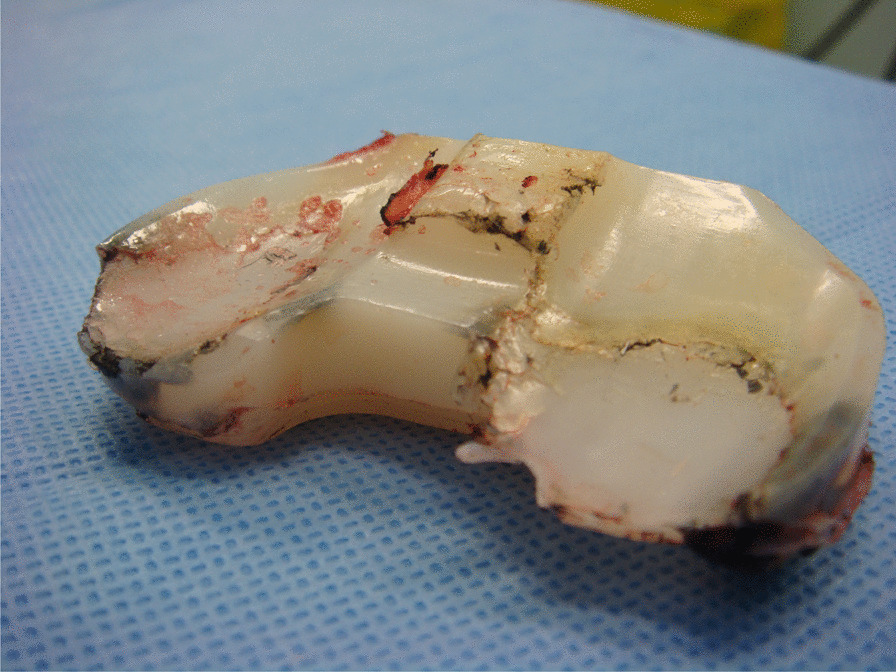
This tibial insert demonstrates catastrophic posterior wear patterns in a patient who underwent a revision for flexion instability

Early posterior stabilised TKRs with flexion instability presented with a vague sense of instability or a cam-post dislocation. More modern designs have increased the cam-post jump distance and dislocation rates are well below 0.5% today [18]. Interestingly, most patients who present with a dislocated PS TKR report it occurring while trying to put a shoe or sock on the operated limb. This position places the leg in deep flexion and applies a varus stress to the knee and so indicates that there may be combined lateral ligamentous laxity and flexion–extension mismatch [4]. Patients at risk may be those who have had a PS TKR and lateral releases performed for a valgus deformity [4, 18, 38]. One must be aware of intra-operative errors that may lead to a flexion–extension gap mismatch and instability. The production of symmetric and balanced gaps is paramount [38, 39]. In 2014 Abdel et al. published their series of 60 patients who underwent revision for flexion instability, and were able to identify factors that led to instability and the degree of correction required re-operatively [16]. They then assessed outcomes using radiographic measurements and Knee Society Scores postoperatively at a mean follow-up of 3.6 years. A mean decrease in posterior condylar offset of 4 mm, distalisation of the joint line and an increased posterior slope by 5° was present in their patients pre-operatively. Upon addressing these issues there was a significant improvement in the mean knee society scores and no reports of instability [16]. Some authors have suggested there may be a limited role for non-operative measures (such as quadriceps strengthening and use of a brace) to treat mild cases of flexion instability, but there is very little published evidence for solid recommendations [16, 40]. Given the success with revision surgery we would recommend this for suitable patients. There is a temptation to simply insert a thicker polyethylene liner, but this would tighten both the extension and flexion space, risk a flexion contracture occurring in the long run, and it is generally not recommended [4, 16, 38–40]. Our treatment of choice follows the step-wise recommendations by Abdel et al. and others [4, 16, 22, 23, 39]. In general, the mainstay of revision surgery is to increase the posterior condylar offset through a larger femoral component. We find that comparison with the native pre-operative condylar offset is a useful guide during planning. The tibial insert typically demonstrates posterior wear (Fig. 3). Addressing the posterior tibial slope, any component malrotation or axial malalignment is essential. Posterior condylar augments may be necessary in cases of excessive posterior condylar resection. In cases where a gap mismatch still exists following upsizing and tibial slope correction, it is recommended to equalise the gaps by further resection of the distal femur and then inserting an appropriate tibial liner. This will raise the joint line and was required in 56% of cases in Abdel et al.’s series which had resulted in stability being restored. When selecting implants, one must understand that the PCL is likely to be deficient in function given the laxity in the flexion space, and so a modern posterior stabilised implant would be recommended in line with most published series [4, 16, 22, 23, 39]. There should be a low threshold to convert to a more constrained implant, as it has been suggested that collateral insufficiency may be present in combination with flexion instability and so careful and regular intra-operative assessment is required to guide implant choices [4, 38].

### Mid-flexion instability

In 1990, Martin and Whiteside conducted a cadaveric TKR study where medial and lateral forces were applied to a joint to simulate varus and valgus stress at 0°, 30°, 45°, 60° and 90° of flexion. They identified mid-flexion laxity when the femoral component was positioned 5 mm proximally and anteriorly. It has been suggested that elevating the joint line would alter the flexion–extension axis and cause laxity in the PCL, collateral ligaments and posterior capsule throughout the mid-flexion range despite the extremes of flexion and extension having symmetric and equal gaps [40, 41]. Elevating the joint line changes the axis of rotation and brings the origin and insertion sites of the collateral ligaments closer to each other. This may cause a pronounced laxity at the mid-flexion ranges of motion (Fig. 2) [19, 22, 42]. The typical scenario that leads to an elevation of the joint line is when a flexion contracture is present and the surgeon completes an excessive distal femoral cut to achieve full extension as opposed to completing a posterior capsular release and posterior osteophyte removal [42]. It is commonly quoted that joint line elevation should not exceed 8 mm of the pre-operative position [43, 44]. This is based on work by Figgie et al. which showed better Mayo Clinic knee scores if the joint line was not elevated beyond 8 mm and a study by Snider et al. which showed lower knee society scores for patients who had their joint lines elevated beyond 8 mm in primary TKRs [43, 44]. Much of the data available were obtained from cadaveric studies, and a body of surgeons questioned the existence and relevance of mid-flexion instability as an isolated instability. However, increased AP laxity in mid-flexion correlated with poorer patient reported outcomes and a subjective feeling of instability [45, 46]. In our practice, we advocate soft tissue releases to address a fixed flexion contracture of the knee in primary TKR, and at revision scenarios for mid-flexion instability we advocate the restoration of the joint line with the use of distal femoral augments if previously elevated. The design of the femoral component has been brought into question as a possible aetiological factor for mid-flexion instability. In 2013, Clary et al. demonstrated a paradoxical anterior femoral translation in mid-flexion with a multi-radius femoral component [47]. They recommended that gradually reducing radius of curvature best addressed this reduction in mid-flexion stability and increased conformity. Wang et al. compared biomechanics during sit-to-stand movements for multi-radius versus single-radius knee designs. They reported a temporary varus–valgus instability during knee flexion in multi-radius femoral components which was felt to occur from a loss of tension in the collateral ligaments [48]. They also reported increased hamstring activation at mid-flexion which was postulated to occur as a technique to compensate for mid-flexion instability [49].

In contrast to this Stoddard et al. were unable to show a significant difference between single-radius and multi-radius TKRs in anterior drawer or stability between 30° and 90° of flexion [50]. Jo et al. assessed stability using a navigation system throughout the range of motion of the knees [51]. They found significantly greater intra-operative stability at 30° of flexion with single-radius knees compared to multi-radius designs though at 2-year follow-up there were no postoperative differences in outcomes [51]. Evangelista et al. sought to determine whether a posterior stabilised TKR versus a CR TKR would lead to greater mid-flexion instability [52]. Their study was unable to demonstrate a significant difference between the two implants, but Hino et al. found a greater degree of varus–valgus laxity between 10° and 90° of flexion with posterior stabilised TKRs compared to CR TKRs [52, 53]. Long-term outcome studies have not diagnosed mid-flexion instability in both posterior stabilised TKRs and CR TKRs with multi-radius components, though this should be interpreted with caution, as it may well result from under-recognition of mid-flexion instability (39, 42, 51–54). Overall, there is a need for further clinical studies into femoral component design or implants and mid-flexion instability to be able to make any reliable conclusions. The principles of managing an unstable TKR do apply, and when mid-flexion instability is identified, one should aim to restore the joint line and balance the gaps.

### Global multiplanar instability

**Fig. 4:**
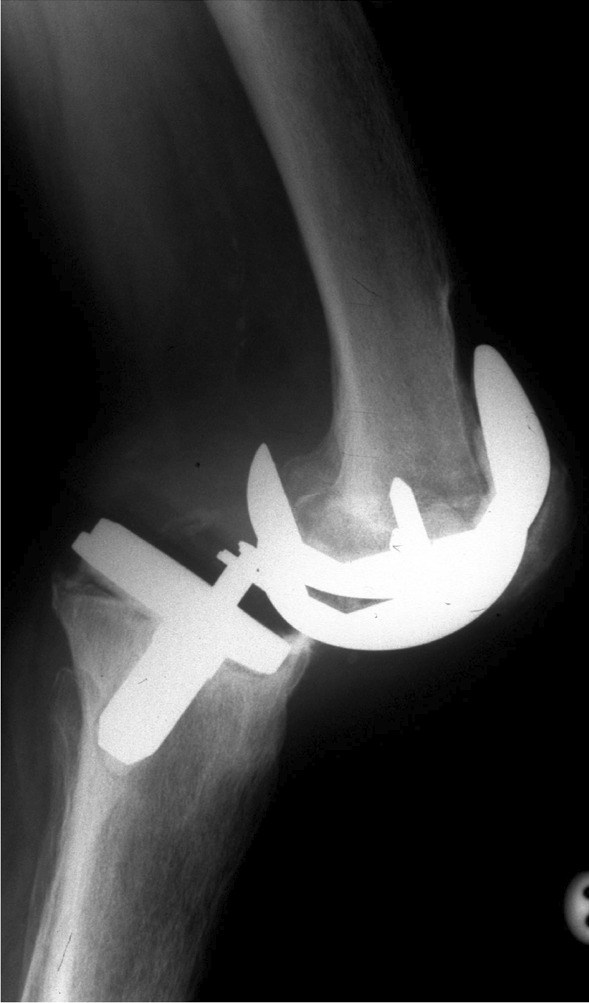
This lateral radiograph demonstrates significant loosening and collapse. The trigger for this presentation was flexion instability which progressed to this presentation. This underlines the importance of early recognition of instability and addressing the appropriate cause

Global instability is instability in more than one plane and is considered a challenge to treat. The patients have usually had multiple operations and present with attenuated tissues and large gap imbalance. When clinically assessing them, the examiner will find multidirectional laxity and often a prosthesis with increased constraint is required to salvage the situation (Fig. 4).

## Conclusions

**Fig. 5:**
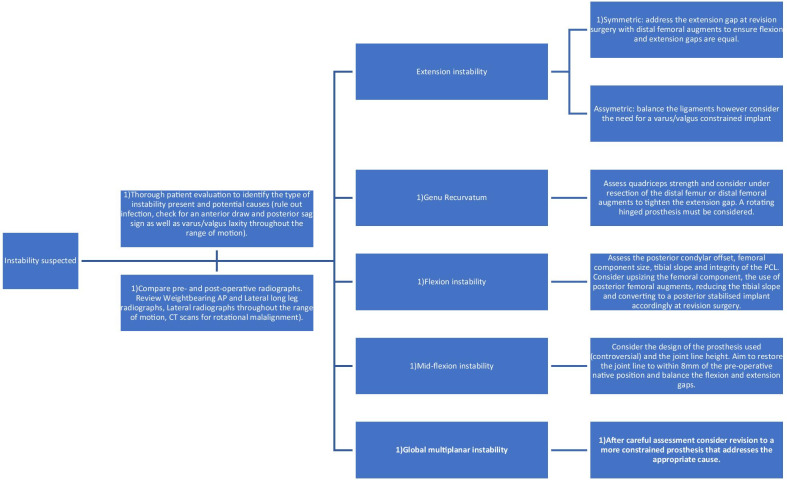
Flow chart summarising management strategies for instability in total knee replacements

Instability constitutes a significant proportion of early failures in total knee surgery. It provides a challenge to the surgeon both in avoiding the surgical errors that produce instability, but also in the diagnosis and resolution of instability as a cause of dissatisfaction in patients with total knee replacement. A summary of treatment strategies has been provided (Fig. 5).

Currently just under 20% of the reasons for a single-stage revision, instability as a cause of dissatisfaction is set to increase as the orthopaedic community and younger, more active patients demand more functional capabilities from their knee surgery. Focus is shifting from survivorship as the measure of success in total knee replacement, to that of functional capability and patient satisfaction.

Orthopaedic surgeons should be fully knowledgeable of the surgical errors that can produce instability and the techniques to avoid that occurrence. They should also be aware of the design attributes of different total knee implants and the effect of different design features that may or contribute to instability.

Diagnosis of functional instability represents a challenge, as there is no diagnostic test other than a raised awareness of the clinical presentations of an unstable total knee replacement, aspects of the history of the patient’s recovery and clinical examination of the knee joint.

Understanding the presentation of instability in total knee replacement is important, as is the classification of the types of instability included in this review. The relevance is that different types of instability will present in different ways, and, once appropriately classified, each type of instability will require a different surgical solution.

Understanding this complex, multifactorial complication of knee surgery along with the methods of its resolution is an essential part of the skill set of specialist knee surgeons.

## Abbreviations

AP: Anterior–posterior
CT: Computed tomography
CR: Cruciate retaining
EDS: Ehlers-Danlos syndromes
ITB: Iliotibial band
LCL: Lateral collateral ligament
MCL: Medial collateral ligament
mm: Millimetres
NJR: National Joint Registry
NICE: National Institute for Health and Clinical Excellence
PJI: Periprosthetic joint infections
PCL: Posterior cruciate ligament
QALY: Quality-adjusted life years
RCT: Randomised Controlled Trial
TKRs: Total knee replacements
UK: United Kingdom
USA: United States of America
